# Raman Spectroscopy, X-ray Diffraction, and Scanning Electron Microscopy as Noninvasive Methods for Microstructural Alterations in Psoriatic Nails

**DOI:** 10.3390/molecules26020280

**Published:** 2021-01-08

**Authors:** Anca E. Chiriac, Doina Azoicai, Adina Coroaba, Florica Doroftei, Daniel Timpu, Anca Chiriac, Mihaela Pertea, Elena-Laura Ursu, Mariana Pinteala

**Affiliations:** 1 Faculty of Medicine, “Grigore T. Popa” University of Medicine and Pharmacy, 700115 Iași, Romania; chiriancut@yahoo.com (A.E.C.); doina.azoicai@gmail.com (D.A.); pertea_mihaela@yahoo.com (M.P.); 2 Centre of Advanced Research in Bionanoconjugates and Biopolymers, “Petru Poni” Institute of Macromolecular Chemistry, 700487 Iași, Romania; adina.coroaba@icmpp.ro (A.C.); florica.doroftei@icmpp.ro (F.D.); ancachiriac@yahoo.com (A.C.); pinteala@icmpp.ro (M.P.); 3 Photochemistry and Polyaddition Department, “Petru Poni” Institute of Macromolecular Chemistry, 700487 Iași, Romania; dtimpu@icmpp.ro; 4 Department of Dermatophysiology, “Apollonia” University, 700511 Iași, Romania; 5 Department of Dermatology, Nicolina Medical Center, 700613 Iași, Romania; 6 Clinic of Plastic and Reconstructive Microsurgery, “Sf. Spiridon” Emergency Hospital, 700111 Iași, Romania

**Keywords:** nail psoriasis, Raman spectroscopy, X-ray diffraction, scanning electron microscopy (SEM)

## Abstract

Psoriasis is a chronic inflammatory disease associated with immune system dysfunction that can affect nails, with a negative impact on patient life quality. Usually, nail psoriasis is associated with skin psoriasis and is therefore relatively simple to diagnose. However, up to 10% of nail psoriasis occurs isolated and may be difficult to diagnose by means of current methods (nail biopsy, dermoscopy, video dermoscopy, capillaroscopy, ultrasound of the nails, etc.). Since the nail is a complex biological tissue, mainly composes of hard α-keratins, the structural and morphological techniques can be used to analyze the human fingernails. The aim of this study was to corroborate the information obtained using Raman spectroscopy with those obtained by scanning electron microscopy (SEM) and X-ray diffractometry and to assess the potential of these techniques as non-invasive dermatologic diagnostic tools and an alternative to current methods.

## 1. Introduction

Psoriasis is a chronic inflammatory disease associated with immune system dysfunction and is highly influenced by both environmental and genetic factors. It is characterized by a hyperproliferation and differentiation of keratinocytes due to an abnormal immune response [1]. Its prevalence is ~2–3% of the world population with equal sex incidence and considerable differences among geographic regions and demographic characteristics [1,2,3]. Approximately 10–78% of psoriasis patients have nails affected, with 5–10% of patients having isolated nail psoriasis [4]. Psoriasis can affect fingernails and toenails, but fingernails are more commonly affected due to the fact that they grow faster [5]. Psoriasis is characterized by a variety of both specific and suspicious clinical manifestations concerning the nail matrix (pitting, leukonychia, crumbling, red spots in the lunula) and the nail bed (salmon patches, hyperkeratosis, onycholysis, splinter hemorrhages) [1,5]. Although nail diseases are considered to be minor, they have a negative influence on all aspects of patients’ quality of life. Nail psoriasis can have physical and psychosocial consequences on patient social life, as patients with nail dystrophies often withdraw from public life due to the disgraceful appearance, and also due to pain, discomfort, and risk of superinfection [6,7].

Usually, nail psoriasis is associated with skin psoriasis and is therefore relatively simple to diagnose. However, up to 10% of nail psoriasis occurs isolated and may be difficult to diagnose. In these cases, various methods are available to confirm the diagnosis: nail biopsy, dermoscopy, video dermoscopy, capillaroscopy, ultrasound of the nails, and optical coherence tomographyand confocal laser scanning microscopy [1]. The above-mentioned techniques are invasive, difficult to perform, less reliable, and expensive, and therefore the development of non-invasive, precise, and fast alternative methods for health evaluation is needed.

The nail is a complex biological tissue, mainly composes of hard α-keratins [8]. These proteins are highly folded due to stable disulfide covalent bonds and also to Van der Waals interactions, hydrogen bonds, and Coulomb interactions [9]. The disulfide bonds are responsible for the keratin’s high resistance to chemical and enzymatic attacks.

**Figure 1:**
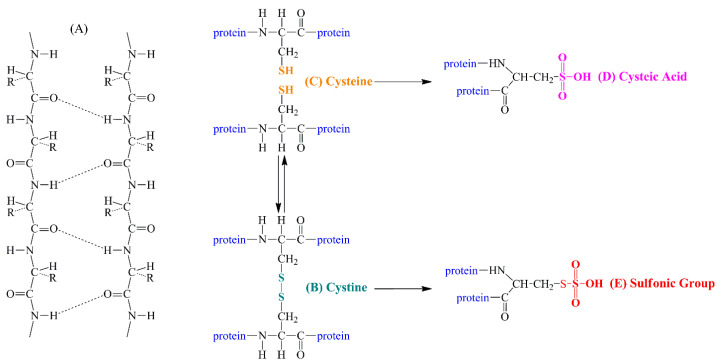
(**A**) Schematic representation of intermolecular hydrogen bonds of keratin structural units. (**B**) Formation of cystine units by oxidation of thiol groups, with the formation of disulfide bridges (–S–S–) in cysteine units. (**C**) Schematic representation of cysteine structural units. (**D**) Schematic representation of cysteic acid units produced as a result of a degradation process under the action of reactive root species occurring in the case of oxidative stress caused by psoriasis. (**E**) Schematic representation of oxidized disulfide units produced as a result of a degradation process under the action of reactive radical species occurred in the case of oxidative stress caused by psoriasis (figure adapted from Coroaba et al. [10]).

The schematic representation of intermolecular hydrogen bonds of keratin structural units is presented in Figure 1A. The keratins contain a significant percentage (24%) cysteine residues (sulfur-rich amino acids) (Figure 1C). By oxidizing the free thiol (SH) groups from the cysteine structure, disulfide bridges (–S–S–) are formed between the protein chains (Figure 1B), offering to keratins the three-dimensional structure, resistance to proteolytic enzymes, and, finally, distinguishing them from other structural nail proteins, such as collagen [10,11]. Therefore, any abnormality in the nails can be a consequence of various diseases, including psoriasis, and it is important to test them in the laboratory [12].

A thorough understanding of the biochemistry, structure, and mechanical properties of keratins would provide useful knowledge of mutations in keratins that can determine a severe disruption of the nail. Previously used techniques to analyze the human nail morphology and structure included Raman spectroscopy [13], X-ray diffraction [14], scanning electron microscopy (SEM) [10], etc. The application of Raman spectroscopy to characterize molecular structures of human nails has been previously reported [13,15,16]. Williams et al. [17] used Raman spectroscopy to study the molecular structure of human keratotic biopolymers (nail, hair, skin), assigning the Raman spectral bands of these materials to specific vibrational modes of the chemical bonds and found that the principal differences in the Raman spectra of these samples are determined by the variations in the sulfur content. Widjaja et al. [16,18] used Raman spectroscopy as an analytical method for gender classification and to differentiate between fingernails and toenails. Raman spectroscopy on nails has been used to establish a relationship between nail composition and bone health. Pillay et al. [19] demonstrated that disulfide bond content is lower in nails collected from the osteoporotic patient compared to the healthy ones. The information obtained through Raman spectroscopy helps to determine the structure or to identify the structural changes that may occur in the analyzed sample under the action of stress factors. The results obtained by Raman spectroscopy can be supplemented with information on the morphology of the same samples by scanning electron microscopy (SEM). The SEM technique is non-invasive and provides accurate identification of changes that may occur in the surface morphology of biological samples [20], including protein dynamics [21], cell study [22], or organ fragments—small multicellular organisms on a micrometric (10−6 m) and nanometric (10−9 m) scale [23]. For example, SEM was used to evaluate the degree of nail damage in onychomycosis and also for preliminary identification of the involved pathogen, when the traditional mycological methods are negative [24,25]. X-ray diffraction was previously used to study the molecular structure of hard α-keratin from different sources, such as hair, wool, etc. [26,27,28]. Saengkaew et al. [29] used X-ray diffraction to study the human-hair microstructures and to establish a relationship between the modifications that occurred in fibrous microstructures with the health-state, for the particular case of breast cancer. Even though hard α-keratin gives X-ray scattering patterns that provide important structural information, there are few reports concerning the study of nail structure by X-ray diffraction.

Because keratin is the main nail protein that does not undergo enzymatic changes, it can be studied in terms of determining changes that may be due to stressors in the body (i.e., overproduction of reactive radical species) and that may influence conformational and structural profile specifically [11,12]. Also, the diagnosis of psoriatic nails can be established without difficulty in a patient with cutaneous psoriasis, but in patients with isolated psoriatic nails or patients with a suspicious diagnosis, several types of investigations should be undertaken. For these reasons, in the present study, the information obtained with Raman spectroscopy was corroborated with SEM and X-ray diffractometry in order to explore the chemistry of keratins, in particular, of keratin degradation due to a specific inflammatory process and to assess the potential of the mentioned techniques as non-invasive dermatologic diagnostic tools and an alternative to the current methods.

## 2. Results and Discussion

### 2.1. Raman Spectroscopy

**Figure 2:**
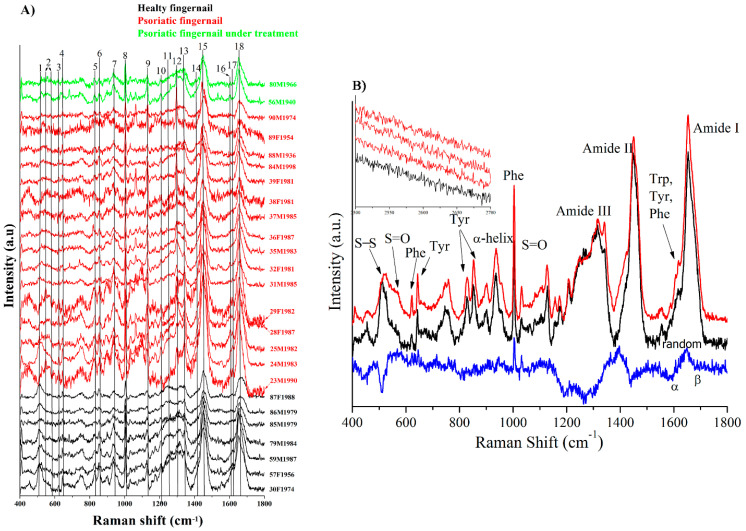
(**A**) The Raman spectra recorded of fingernail clippings: healthy (black), psoriatic (red), and psoriatic under treatment from (green). (**B**) The subtracted Raman spectra (blue) of the psoriatic (red) from healthy fingernails (black). Inset: the 2500 ÷ 2700 cm^−1^ region for healthy (black) and psoriatic fingernails (red).

The Raman spectra provided information on the functional groups of the fingernails, allowing a comparison of the chemical composition between healthy and psoriatic ones. Figure 2A shows the Raman spectra recorded from fingernail clippings of normal and psoriatic patients together with the ones under treatment with adalimumab. Several key features can be pointed out after a close introspection of the Raman spectra of healthy and psoriatic fingernail clippings and the residue obtained after the subtraction of the Raman spectra of the psoriatic nail from the healthy ones (Figure 2B). If the band is negative in the subtracted spectrum, it indicates that the bond content from healthy fingernails is decreasing in psoriatic fingernails and if the band is positive, vice versa as before.

The Raman band centered at 515 cm^−1^ (Figure 2) is assigned to the S–S stretching mode (gauche–gauche–gauche conformation of the C–C–S–S–C–C moiety) of cystine residues (Figure 1C). Disulfide bridges are particularly interesting since they are directly related to the microstructure of the nail and the disulfide cross-linking is determinant for the physical and mechanical properties of keratin. The ratios of the peak areas corresponding to S–S bond (515 cm^−1^) to that of C–C bond (936 cm^−1^) for healthy and psoriatic fingernails were determined as 1.01 and 0.64, respectively. This finding suggests a significant decrease of disulfide bridges in psoriatic fingernails that can also be seen in the negative band of the subtracted spectrum (Figure 2B, blue color). This was previously observed also by Gómez et al. [30].

The decreasing of the peak area of S–S bond is accompanied by the appearance of a shoulder at around 540–570 cm^−1^ for psoriatic fingernails, assigned to the vibration mode of –S–S=O or –S=O bonds, suggesting the formation of sulfur-containing oxidation products (Figure 1D,E). This is also confirmed by the increased intensity of the 1025 cm^−1^ peak (positive peak in the subtracted spectrum), due to the stretching vibrations of –S=O moiety (Figure 2). It can be concluded that the psoriasis presence in the body is acting as a strong oxidizing factor, producing the degradation of the disulfide bridges, with the loss of the partial integrity of the keratin structure, as observed before by our group through FT-IR and XPS investigations [10].

Tyrosine and tryptophan residues from the protein sequence determine several bands in the Raman spectrum of α-keratin. The weak Raman bands at 623 cm^−1^ and 643 cm^−1^ (Figure 2) can be assigned to the C–S stretching vibration of cystine and cysteine residues.

Another important indication in the evaluation of the nail degradation is the monitoring of the ratio between the peak areas of the bands at 830 cm^−1^ and 850 cm^−1^ (I830/I850) which corresponds to the presence in the keratin of unmodified and denatured tyrosine, respectively, or the changes of its location [31]. When the I830/I850 ratio in the psoriatic fingernails decreases compared to the healthy ones, it can be concluded that under the action of aggressive factors, such as the appearance of the overproduction of reactive oxygen radicals (ROS), a situation favored by the existence of psoriasis, tyrosine is degraded or changes its location in a much more hydrophobic area. In the case of the analyzed samples in this study, the I830/I850 ratio was in the range of 2.1 ÷ 1.4 for healthy fingernails, and in the nails affected by psoriasis, the ratio was between 1.4 and 0.4. The decrease of this ratio indicates that psoriasis generates an overproduction of ROS causing degradation of the nail bed due to the presence of psoriasis in the body [31].

The Raman band at 1003 cm^−1^ is associated with the symmetric breathing mode of the aromatic ring of phenylalanine. The broadband at 1200 ÷ 1400 cm^−1^ is determined by the overlapping of Raman bands corresponding to the C–H deformation and amide III. Amide II (1450 cm^−1^) involves the deformation of C–H, CH_2_, and CH_3_ moieties. It can be also noted that in the subtracted spectrum, the negative peaks appear in the specific areas for the amide III (1206 ÷ 1305 cm^−1^) and amide I (1642 ÷ 1670 cm^−1^) regions, being additional arguments for demonstrating the action of psoriasis in the studied tissue degradation. In this context, the band at 1652 cm^−1^ can be assigned to the amide I vibrational mode (C=O stretching vibrations, C–N stretching, Cα–C–N bending, and N–H in-plane bending of peptide groups). This region reflects the changes in the secondary structures (β-sheet, random coil, α-helix, and β-turns). A slight reduction of α-helix content and an increase of random coil content were observed for psoriatic fingernails. The α-helix is responsible for the stability of the nail proteins, and a decrease of its content suggests destabilization, while an increase in the random coil content indicates the protein denaturation [32].

Gómez et al. [30] and Baraldi et al. [33] reported the presence of a band at about 2550 ÷ 2600 cm^−1^ in the Raman spectrum characteristic to the cleaved disulfide bond into thiol residue (Figure 1D) for onychomycotic nails. This band was not present in our Raman spectrum (Figure 2B, inset), suggesting the possibility to discriminate between two nail diseases by Raman investigations when the diagnostic is uncertain.

**Figure 3:**
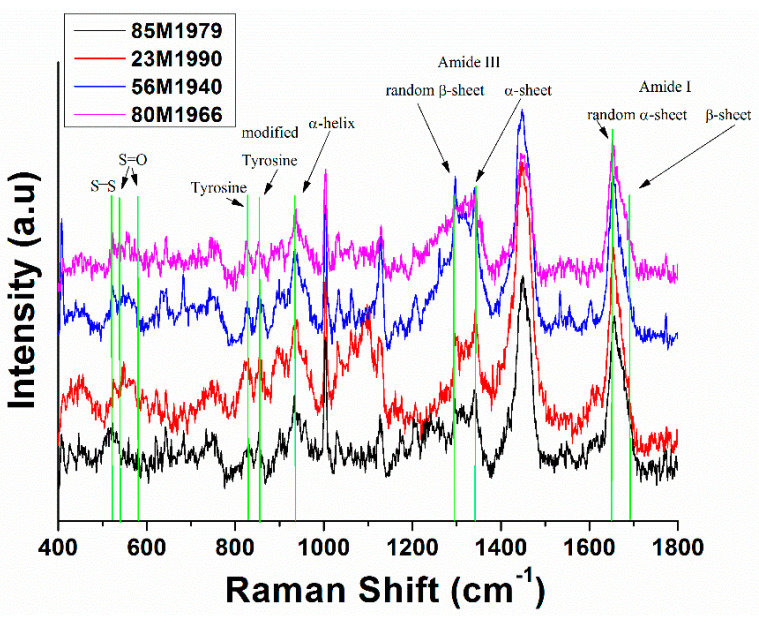
Raman spectra of fingernail clippings from a healthy donor (black), a patient diagnosed with psoriasis (red), and two patients diagnosed with psoriasis and treated with adalimumab (blue and purple).

On the other hand, the analysis of samples from two patients diagnosed with psoriasis and that undergo biological treatment with adalimumab were taking into consideration (Table 1 from Materials and Method Section, 80M1979 and 56M1940). Figure 3 shows the Raman spectra corresponding to samples from a healthy patient (Table 1 from Materials and Method Section, 85M1979), one patient diagnosed with psoriasis (Table 1 from Materials and Method Section, 23M1990), and two patients diagnosed with psoriasis but under adalimumab treatment (Table 1 from Materials and Method Section, 56M1940 and 80M1966). We mention that all the samples came from men of different ages. From Figure 3, it can be seen that the control sample shows a significant peak corresponding to the S–S bond (510 cm^−1^), while the peaks corresponding to the samples from the psoriatic patients are smaller, including in the samples corresponding to the patients under treatment. At the same time, it can be seen that the peaks in the 530–570 cm^−1^ region significantly increase in the samples from patients diagnosed with psoriasis, which means that there is an oxidation of the S–S bridges. There is also a slight increase in the corresponding S–S peak of treated nails corresponding to the patients undergoing treatment with adalimumab compared with a healthy one, leading to the conclusion that adalimumab treatment helps to restore the S–S bridges.

Differences can also be observed in other regions of the Raman spectra, such as in the area of the peaks at 830 cm^−1^ and 850 cm^−1^, corresponding to tyrosine and modified tyrosine, respectively. In this case, an increase in the ratio of peak areas from 830 cm^−1^ and 850 cm^−1^ can be observed for the 80M1966 sample, this ratio becoming higher than 1 compared to that for the patient without treatment (0.4). Moreover, the peak profiles corresponding to amide III and amide I are improved in treated psoriatic nails as compared with untreated nails, becoming more similar to the profile corresponding to the healthy nails. This observation is an additional argument to conclude that adalimumab treatment contributes to the restoration of the keratin structure.

### 2.2. X-ray Diffraction Characterization

**Figure 4:**
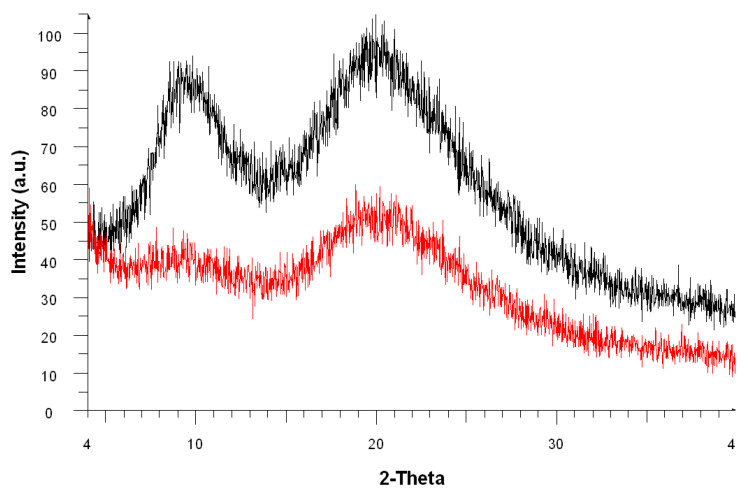
X-ray diffractogram (XRD) patterns of normal (red) and psoriatic fingernails (black).

According to previous reports [26,28,29], hard α-keratin presents two characteristic bands in X-ray diffractogram (XRD): first, around 9°, corresponding to the distance between α-helical axes and the second, around 20°, which refers to the periodic-distance of the 3.6-alpha-helix structure. For both psoriatic and healthy fingernails, the α-keratin characteristic bands can be observed, but these are significantly weaker and slightly shifted to smaller angles for the psoriatic one (Figure 4). Minor shifting between the bands in our samples is an indicator of differences between crystalline domains of the keratin. Furthermore, the crystallinity index of psoriatic fingernail’s keratin decreased (22.6% for psoriatic fingernail compared with 34.6% for normal fingernail), and that suggests the probability of the α-helix crystal structure being partially destroyed by the disease (Table 2). These findings are in agreement with the results obtained through Raman spectroscopy.

### 2.3. Scanning Electron Microscopy

**Figure 5:**
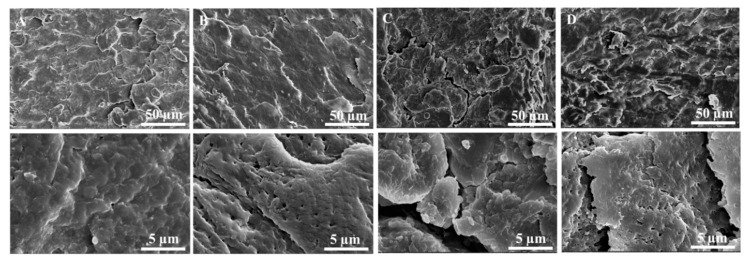
Scanning electron microscopy (SEM) micrographs of the dorsal surface of healthy (column (**A**,**B**)) and psoriatic fingernails (column (**C**,**D**)).

The differences found between healthy and psoriatic fingernail clippings are clearly illustrated by SEM images (Figure 5). Micrographs were taken at two different magnifications in order to easily evaluate the characteristics of both types of fingernail clippings and to highlight the fractures on their surface. The SEM micrographs for the dorsal surface of healthy fingernails displayed a dense, compact, relatively smooth surface, with occasional fractures [34]. For the psoriatic fingernails, it can be seen that the roughness of the dorsal surface is higher compared with healthy fingernails and also, the presence of fragmented regions, with more irregular surface, probably associated with the breaking of disulfide bridges and conformational changes of keratin.

## 3. Materials and Methods

### 3.1. Materials

Fingernail clippings samples were obtained from healthy volunteers (N = 7), with no drug intake or local nail treatment and from patients diagnosed with nail psoriasis (N = 18, from which 16 non-treated and 2 patients treated with biologics) (Table 1). Persons enrolled in the study were given a number, becoming anonymously and data were collected, such as age, gender, body mass index (BMI) weight (kg)/height (m)^2^, personal and familial medical history. Diagnosis of psoriasis was based on clinical and histologic features; for each patient, Psoriasis Area and Severity Index (PASI) and Nail Psoriasis Severity Index (NPSSI) were calculated. Written informed consent was obtained from each individual enrolled in the study. The study was approved by the Ethics Committee of “Grigore T. Popa” University of Medicine and Pharmacy, following the Declaration of Helsinki protocols. From each person, fingernail clippings were obtained and labeled as samples. All samples were washed with acetone, rinsed five times with distilled water to remove contaminants, dried at room temperature, and then stored in an Eppendorf tube at room temperature.

### 3.2. Methods

#### 3.2.1. Raman Spectroscopy

Raman spectroscopy was performed on healthy and psoriatic fingernail clippings using an InVia Raman microscope (Renishaw, Wotton-under-Edge, UK) in the 400–1800 cm^−1^ range. The Raman scattering was excited with a 785 nm near-infrared diode laser. The fingernails were placed with the dorsal surface faced at the laser beam. For each fingernail, spectra were taken for five randomly selected locations to ensure statistical variability (no significant changes in the spectral features were observed). Since the raw spectra acquired from the fingernail presented a broad autofluorescence background, all the spectra were subjected to baseline correction using WiRE 3.2 software(Renishaw, Wotton-under-Edge, UK). Then, each spectrum was subjected to normalization using the Amide I band, 1652 cm^−1^ as the reference peak for a better spectral comparison between the healthy and psoriatic samples.

#### 3.2.2. Scanning Electron Microscopy

Healthy and diseased fingernail surface morphology was examined with a Verios G4 UC Scanning Electron Microscope (Thermo Fisher Scientific, Waltham, MA, USA) at 30 kV. The samples were platinum coated and fixed with double adhesive tape on cylindrical Al conducting supports. Images from the dorsal surface of the fingernail were taken at randomly selected locations.

#### 3.2.3. X-ray Diffraction

X-ray diffraction characterization of the samples was carried out using a D8 ADVANCE (Bruker AXS, Karlsruhe, Germany) device, using the Cu-Kα radiation (λ = 0.1541 nm) and a parallel beam with Göbel mirror. The working conditions were 36 kV and 40 mA, in stepwise mode, (count time 4 s/step, step size 0.02°). The crystallinity index (C.I.) was calculated using the L. Segal formula [35]:

(1) $$C.I=\left(I_{9}-I_{14}\right)/I_{9}$$

where I_9_ is the maximal intensity of crystal lattice diffraction with 2θ ~ 9°, and I_14_ is the minimum intensity of crystal lattice diffraction with 2θ ~ 14°.

## 4. Conclusions

Raman spectroscopy, scanning electron microscopy (SEM), and X-ray diffractometry were used to study the microstructure of healthy and psoriatic fingernail clippings. The results obtained by Raman spectroscopy and X-ray diffraction are complementary and suggest that the α-helix structure of keratin is partially destroyed by the presence of psoriasis. The SEM images illustrate that psoriasis affects the surface morphology of healthy nails in terms of uniformity, density, and roughness that can be correlated with the conformational changes of keratin. We have to point out that the peak at 2550 ÷ 2600 cm^−1^ in the Raman spectra, which is responsible for the SH groups and which was observed in the Raman spectra of nails with onychomycosis [30,33], is not present, leading to the conclusion that psoriasis is much more aggressive than the presence of onychomycosis, acting on the S–S bond through sulfonic groups formation. Also, under biological treatment, the reformation of –S–S– bridges is observed. In conclusion, our findings indicate that the common techniques used for structural and morphological characterization could help in the development of reliable and noninvasive dermatologic diagnostic methods.

## Figures and Tables

**Table 1:** A brief presentation of the healthy donors or patients from whom the fingernail clippings samples were taken. (M: male; F: female).

	Sample Code	Sex	Age
Healthy volunteers	87F1988	F	32
	86M1979	M	41
	85M1979	M	41
	79M1984	M	36
	59M1987	M	33
	57F1956	F	64
	30F1974	F	46
Non-treated psoriatic patients	90M1974	M	46
	89F1954	F	66
	88M1936	M	84
	84M1998	M	22
	39F1981	F	39
	38F1981	F	39
	37M1985	M	35
	36F1987	F	33
	35M1983	M	37
	32F1981	F	39
	31M1985	M	35
	29F1982	F	38
	28F1987	F	33
	25M1982	M	38
	24M1983	M	37
	23M1990	M	30
Biologics-treated psoriatic patients	80M1966	M	54
	56M1940	M	80

**Table 2:** X-ray diffractogram (XRD) characteristics of the healthy and psoriatic fingernails.

Sample	2θ (°)	D Value (Å)	Intensity %	C.I. (%)
Healthy fingernail	9.7	9.1	82.5	34.6
	20.2	4.4	100	
Psoriatic fingernail	9.4	9.4	55.7	22.6
	19.8	4.5	100	

## Data Availability

Restrictions apply to the availability of the data presented in this study because patients did not provide permission for data sharing outside the institution or established collaborations.
